# Notes from Dental Practice

**Published:** 1868-12

**Authors:** 


### ARTICLE III.

### *Notes from Dental Practice.*

Case I.—Irregularity. The superior central and lateral incisors overlapping; right superior cuspid to the inside of arch and striking on the inside of the inferior teeth; left superior cuspid projecting so far as to cause deformity of the lip.

History.—Patient, a female aged 17 years. Cause of irregularity—a contracted arch and teeth of large size.

Treatment.—Extracting the superior right and left first bicuspids gave space. A band, cut from rubber tubing, passing from the left superior cuspid to the first molar on the same side, moved the projecting cuspid to the position formerly occupied by the first bicuspid. A silk ligature confined this tooth during the time a similar band was acting on the left lateral incisor and drawing it back to a normal position.

The same plan was adopted in the case of the left central incisor; the pressure of the lip against the projecting angles of the central and lateral forcing them inwards.

Silk ligatures confined all of the restored teeth of the left side, and attention was then directed to the deviating teeth on the right side.

By means of a narrow curved bar of vulcanized rubber, attached at one extremity to the labial surface of the right central incisor by means of a silk ligature, and at the other to the buccal surface of the first molar on the same side, and over which a band of rubber was stretched which passed around the neck of the cuspid, this tooth, inclining inwards, was brought out to its proper position. To prevent the occlusion of the jaws from interfering with this operation, prior to the application of the curved bar an impression of the grinding surfaces of the right superior and inferior first molars was obtained, and a cap of vulcanized rubber made to keep the teeth of both jaws at such a distance as to allow of the drawing outwards of the deviating cuspid. This cap was worn until the point of the cusp of the deviating tooth was forced to the outside of the inferior teeth. When this was accomplished, the bar and cap were no longer necessary. The curved bar referred to can be readily obtained from the palatine surface of a discarded rubber set of teeth.

The same plan was pursued for restoring the right lateral incisor as that before adopted in the case of the left lateral.

The right central incisor, however, owing to its overlapping so considerably the left central, could only be forced towards the right lateral by means of the rubber band and could not be turned on its axis by the pressure of the lip.

A normal position, however, was gained by a small band of platina plate, so shaped as to fit accurately the crown of the incisor, to which small knobs, made from the headed pins of rubber teeth, were attached ; ligatures being attached to these knobs and acting in opposite directions.

All of the irregular teeth being brought into their proper places, an impression of the mouth was obtained, and a vulcanized rubber plate made to fit accurately the roof of the mouth and also the necks of the posterior teeth, with its anterior edge extending for some distance upon the palatine surfaces of the restored teeth. By such an appliance these teeth were confined in their new position until they became firmly fixed.

Case II.—Alveolar Abscess in a superior lateral, with a fistulous opening on the palate opposite the first bicuspids; the tooth free from caries but greatly discolored and causing much annoyance.

History.—Patient, female aged about 30 years. Cause—mechanical violence occurring 18 months before treatment.

Treatment.—An opening was first drilled into the pulp cavity on the palatine surface, and tepid water injected through this opening by means of a syringe; the water mixed with pus and blood making its way into the mouth through the fistulous opening in the palate. This operation was continued until the water passed free from discoloration.

The first medicinal agent used was carbolic acid on a strand of floss silk, which was passed to the apex of the root through the pulp canal; an application of the same agent was made to the fistulous opening in the palate, and the end of the silk projecting from this opening was moistened with sandarach varnish which closed the orifice.

The floss silk in the pulp canal was not confined in this manner, the object being to allow the pus formed to escape through the opening in the palatine surface of the tooth.

An examination twenty-four hours after the first application was made, showed a decided improvement, at least as re garded the direction of the discharge which was now confined to the pulp cavity and opening on the palatine surface. Two more applications of carbolic acid were made in the same manner as the first, followed by one of carbolic acid and tannin, and the gum about the tooth bathed with a preparation composed of equal parts of the tincture of iodine and arnica.

So decided was the improvement after these applications, that the remedial agent was changed to carbolic acid in glycerine on the strand of silk, and the opening in the crown leading to the pulp cavity closed, first by cotton saturated with sandarach varnish, and afterwards by a temporary filling of Hill's stopping. This latter application of carbolic acid in glycerine was renewed every three or four days, and the pulp canal subsequently filled to near the apex with gold as well as the crown cavity, without any return of the symptoms.

Prior to filling the crown cavity, the color of the crown of the tooth was in great measure restored by bleaching with Labarraque's Solution.

Case III.—Bleaching a Necrosed Tooth.

History.—The lateral incisor referred to in Case II. The discoloration very decided, and of a bluish-black color.

Treatment.—After filling about two-thirds of the pulp cavity with gold, the following method was resorted to in order to remove the discoloration from the crown of the tooth:

The crown cavity prior to the introduction of the pulp cavity filling, having been properly prepared, a pellet of cotton saturated with the solution of chloride of soda known as "Labarraque's Disinfecting Liquid" was introduced into the crown cavity and allowed to remain for one hour, when it was removed and a temporary filling of Hill's Stopping inserted.

The patient was dismissed until the following day, as it was not convenient to make repeated applications at one sitting, when a second application of the same agent was made and allowed to remain in the tooth for the same length of time. Some six applications were made in this case, but some improvement in the appearance of the tooth was visible after the second application.

When the discoloration is not so decided as it was in th case, one or two applications of the agent may accomplish the purpose.

From the number of applications made, the color of the tooth was greatly improved and the crown cavity permanently filled with gold. The agent used in this case, Labarraque's Disinfecting Fluid, Liquor Sodæ Chlorinatæ, is prepared from chlorinated lime, carbonate of soda and water, in the proportion of one pound of the lime, two pounds of the soda and a gallon and a half of the water. Medicinally it is used internally in malignant diseases, locally in gangrenous and other ulcers of a malignant character and for ulceration of the gums; it is also a powerful disinfectant.

Case IV.—Epulis or Fibrous Tumor of the Gums, extending from the left superior lateral incisor to the second molar of the same side, and causing considerable deformity owing to its protruding the lip.

History,—Some six months before treatment, this morbid growth made its appearance on the free surface of the gum, between the necrosed roots of the first and second superior bicuspid teeth, causing little or no pain, except when irritated by the contact of hard substances.

Treatment.—The necrosed roots giving rise to this morbid growth were first removed. The tumor having a broad base, a double silk ligature was introduced through the centre of the base and as close to the alveolar process as was possible. One thread of this ligature was then tied tightly around the posterior half of the base, and the other thread about the anterior half, in order that sloughing might occur from the cutting off of its vascular supply.

On examining the tumor several days after the application of the ligature, it was determined owing to the dark purple appearance it presented and which resulted from the constriction, to remove it at once.

This was readily accomplished with a pair of dissecting scissors, and the hemorrhage, which was considerable, arrested by means of powdered sub-sulphate of iron. This preparation was applied to the wounded surface on a pellet of cotton first saturated with sandarach varnish in order that the powder might adhere to it. The hemorrhage being thus arrested, the remaining portion of the base was removed by means of the scalpel close to the alveolar process, and the surface touched with nitrate of silver.

There has up to this time, six months after the operation, been no indication of the reproduction of the morbid growth.

